# Efficacy of umbilical cord cleansing with a single application of 4% chlorhexidine for the prevention of newborn infections in Uganda: study protocol for a randomized controlled trial

**DOI:** 10.1186/s13063-017-2050-0

**Published:** 2017-07-12

**Authors:** Victoria Nankabirwa, Thorkild Tylleskär, Josephine Tumuhamye, James K. Tumwine, Grace Ndeezi, José C. Martines, Halvor Sommerfelt

**Affiliations:** 1 Department of Epidemiology and Biostatistics, School of Public Health, College of Health Sciences, Makerere University, PO Box 7072, Kampala, Uganda; 2 Centre for Intervention Science in Maternal and Child Health, Centre for International Health, University of Bergen, Bergen, Norway; 3 Department of Paediatrics and Child Health, School of Medicine, College of Health Sciences, Makerere University, Kampala, Uganda; 4 Global Women and Children’s Health, Norwegian Institute of Public Health, Oslo, Norway

**Keywords:** Chlorhexidine, Omphalitis, Newborn, Severe illness, Neonatal, Trial

## Abstract

**Background:**

Yearly, nearly all the estimated worldwide 2.7 million neonatal deaths occur in low- and middle-income countries. Infections, including those affecting the umbilical cord (omphalitis), are a significant factor in approximately a third of these deaths. In fact, the odds of all-cause mortality are 46% higher among neonates with omphalitis than in those without. Five large randomized controlled trials in Asia and Sub-Saharan Africa (SSA) have examined the effect of multiple cord stump applications with 4% chlorhexidine (CHX) for at least 7 days on the risk of omphalitis and neonatal death. These studies, all community-based, show that multiple CHX applications reduced the risk of omphalitis. Of these trials, only one study from South Asia (the Bangladeshi study) and none from Africa examined the effect of a single application of CHX as soon as possible after birth. In this Bangladeshi trial, CHX led to a reduction in the risk of mild-moderate omphalitis and neonatal death. It is important, in an African setting, to explore the effect of a single application among health-facility births. A single application is programmatically much simpler to implement than daily applications for 7 days. Therefore, our study compares umbilical cord cleansing with a single application of 4% CHX at birth with dry cord care among Ugandan babies born in health facilities, on the risk of omphalitis and severe neonatal illness.

**Methods:**

The CHX study is a facility-based, individually randomized controlled trial that will be conducted among 4760 newborns in Uganda. The primary outcomes are severe illness and omphalitis during the neonatal period. Analysis will be by intention-to-treat.

**Discussion:**

This study will provide novel evidence, from a Sub-Saharan African setting, of the effect of umbilical cord cleansing with a single application of 4% CHX at birth and identify modifiable risk factors for omphalitis.

**Trial registration:**

ClinicalTrials.gov, identifier: NCT02606565. Registered on 12 November 2015.

**Electronic supplementary material:**

The online version of this article (doi:10.1186/s13063-017-2050-0) contains supplementary material, which is available to authorized users.

## Background

Ninety-eight percent of the estimated worldwide 2.7 million annual deaths in the first 28 days of life occur in low- and middle-income countries (LMICs) [1]. Nearly a third of these neonatal deaths are associated with infections and this proportion is higher in areas and countries where nearly half of the births occur at home, such as Uganda [2]. Infection of the umbilical cord stump (omphalitis) is a significant contributor to these infections in newborn babies in LMICs [2, 3]. In a Nepalese study, the odds of all-cause mortality were 46% (95% CI 8–98%) higher among infants with umbilical cord infection than in those without [4]. The umbilical cord is cut after birth and the remaining cord stump generally dries and falls off within 5–15 days [3]. Before it detaches, the stump provides necrotic tissue suitable for bacterial colonization, and can act as an entry point for pathogenic microorganisms into the newborn. Newborns are particularly prone to infection, such as omphalitis, for a number of reasons including an immature immune system, exposure to maternal vaginal organisms during birth and unhygienic practices after birth. These practices include cutting of the cord with unsterile instruments, inadequate hand-washing by birth attendants and other caretakers and topical application of harmful substances such ash and cow dung. The increased risk of infection among newborns is even greater for those born preterm who have a more immature immune system and more vulnerable and thinner skin compared to their term counterparts. Several interventions have been recommended in the past to combat newborn infections [2]. These include: full-body skin cleansing with antiseptics like chlorhexidine (CHX); hand-washing with soap and water and use of clean birth kits. But the effects of these interventions on the incidence of infection and death in neonates have been mixed and two systematic reviews did not unequivocally conclude that any of these interventions reduced the risk of either serious infection or death. The World Health Organization (WHO) recognizes that cord care is a crucial component of immediate newborn care. It currently recommends dry cord care for babies born in health facilities in low-income countries while noting that antiseptics could be used in settings where harmful cord practices are rife [5].

Nonetheless, this WHO recommendation of dry cord care for LMIC facility births is questionable given the inadequate evidence on which it is based. The WHO’s recommendation was based on a Cochrane review by Zupan et al. that found no difference in the incidence of umbilical cord infection when topical antiseptics were compared to placebo. With the exception of one study in Thailand, the rest of the 22 trials included in this review were from high-income countries. It would be problematic to generalize these findings to LMICs. There are several potential topical applications that could be used to lower the risk of neonatal infections and death through appropriate cord care in LMICs, such as CHX, povidone iodine, ethanol, gentian violet, silver sulfadiazine and others. Of these, CHX shows the most promise for the following reasons: (1) low cost, (2) broad-spectrum activity against gram-negative and gram-positive bacteria, (3) strong tissue-binding potential with residual effectiveness and (4) a wide safety record. Several studies have shown that CHX reduces cord stump colonization by bacteria and the WHO recommends it as the preferred agent if an antiseptic is to be used on the cord. In addition, CHX is included in the WHO Essential Drug List and is also identified as one of the commodities in the UN commission’s report on life-saving commodities for women and children.

### State of the existing academic literature on topical application of chlorhexidine to the umbilical cord stump

CHX has been used for decades in various health care settings as an antiseptic and, in most of these cases, it has been shown to reduce bacterial colonization and the risk of infection. With respect to newborn cord care in LMICs, two randomized trials in the 1990s of vaginal and newborn washing with 0.25% CHX showed promise but two more recent studies, one in South Africa using 0.5% and the other in Pakistan using 0.6% CHX, did not find a lower neonatal mortality in the intervention group [2]. A more recent approach has been to apply 4% CHX directly to the umbilical cord stump. We found five published reports of large randomized trials, all community-based, that have been done in LMICs; three in Asia and two in Sub-Saharan Africa (SSA) [6–10]. These studies showed that multiple CHX applications may lead to a marked reduction in the incidence of omphalitis but effects of CHX on neonatal mortality vary considerably (Table 1).

**Table 1:** State of the existing academic literature on topical application of chlorhexidine on the umbilical cord stump

		Risk of omphalitis/1000			Neonatal mortality/1000		
Author, Year	Country	Intervention	Control	Adjusted RR	Intervention	Control	Adjusted RR
Mullany, 2006 [6]	Nepal	30.1	62.7	0.46 (0 · 36, 0 · 59)	14.6	19.3	0 · 76 (0 · 55, 1 · 04)
Soofi, 2012 [7]	Pakistan	31.7	75.9	0.58 (0 · 41, 0 · 82)	22.8	36.1	0.62 (0 · 45, 0 · 85)
Arifeen, 2012 [8]	Bangladesh	14.7	26	0.55 (0 · 31, 0 · 95)	26.6	28.3	0.94 (0 · 78, 1 · 14)
Sazawal, 2016 [10]	Tanzania	78.4	115.5	0.65 (0 · 61, 0 · 70)	10.5	11.7	0.90 (0 · 74, 1 · 09)
Semrau, 2016 [9]	Zambia	4.43	6.1	0.73 (0 · 47, 1 · 13)	15.2	13.6	1.12 (0 · 88, 1 · 44)

*RR* risk ratio

With the exception of the Bangladeshi study, all these trials compared at least 7 days of daily CHX application to placebo or dry cord care. The trial in Bangladesh compared a single application of CHX to dry cord care and reported an efficacy of 20% on neonatal mortality (risk ratio 0.80; 95% CI 0.65–0.98) [8]. There was, somewhat surprisingly, no effect of daily cleansing with CHX for 7 days on neonatal mortality.

There is strong evidence for the effect of 7-day CHX application on the risk of omphalitis but the results from the large trial in Bangladesh and the two African trials question whether this translates into a mortality reduction. Daily CHX applications for 1 week have been associated with delayed cord falling which in turn could increase the probability of infection via the cord, and possibly explain the lack of an effect of daily cleansing for 7 days in some studies [11]. A single CHX application could mitigate the reported delay in cord falling when multiple applications are used. There is no data from Africa, and only one study from Asia, that examined the effect of a single application as soon as possible after birth. It is important, in an African setting, to explore the effect of a single application which is also programmatically much simpler to implement than daily application for 7 days. Our study will, therefore, embark on a head-to-head comparison of a single CHX application to dry cord care in birth facilities on omphalitis and severe illness during the neonatal period.

## Study objectives

1. To measure the effect of newborn cord care with a single cleansing of the umbilical cord stump using 4% CHX on the risk of severe illness among Ugandan neonates
2. To measure the effect of umbilical cord cleansing with a single application of 4% CHX at birth on the risk of omphalitis in Ugandan neonates

## Methods/Design

### Study design

This is an individually randomized facility-based controlled trial in which half of the newborns will be randomized to receive the intervention and the other half will receive the standard of care (dry cord care).

### Setting

The trial will be conducted in three health facilities: Mukono Health Center IV, Kawaala Health Center III and Kitebi Health Center III. These three facilities have a monthly combined average of 2400 antenatal visits and 1200 deliveries. Mukono Health Center IV is located within Mukono district while Kitebi and Kawaala Health Centers are located in Kampala district, the capital city of Uganda. Kampala has an estimated resident population of two million while Mukono district, located 25 km from Kampala City has a largely rural population of almost 60,000 people.

### Participants

The study will be carried out among newborns of HIV-1-negative mothers giving birth at the three clinics in Kampala and Mukono districts. Conducting this trial in a homogeneous group of HIV-unexposed (HU) neonates will substantially reduce the risk of imbalance of HIV exposure between study arms and thus mitigate any potential confounding from such an imbalance. Moreover, a finding of CHX protecting babies from severe illness and/or omphalitis among HU neonates can be cautiously generalized to similar populations elsewhere.

### Inclusion criteria

Children born of HIV-1-negative mothers in the three study clinics.

### Exclusion criteria

1. Newborns with severe congenital anomalies
2. Newborns with infection of the umbilical cord when born
3. Severely ill infants requiring hospitalization immediately after birth
4. Children of mothers who cannot appropriately give consent within 12 h of birth
5. Babies born with a birth weight of less than 1500 g

### Randomization

For each of the three health centres, a computer-generated random sequence list with permuted blocks of varying size (4, 6 or 8) was generated by CISMAC scientist Hans Steinsland, who is otherwise not involved in the trial. Based on this sequence, children are randomly allocated to intervention or comparison arms in a 1:1 ratio. Concealment is by a cell phone-based application that yields the trial arm allocation only after consent for inclusion has been confirmed.

### Intervention

The intervention is umbilical cord stump cleansing with a single application of 4% CHX solution at birth. The watery solution was obtained from Galentic Pharma in India (http://www.galentic.com) and came in packages of 10-ml dropper bottles. The application is provided by a trained health worker (nurse or midwife) at the health facility as soon as possible and no later than 12 h after birth. The solution is applied using the dropper bottle onto the tip of the infant’s umbilical cord stump, its base and the area of skin surrounding it. A CHX-moistened cotton ball is then used to gently cleanse the base of the stump and the surrounding skin. Health workers have been trained by general physicians and pediatricians to correctly, consistently and safely apply CHX. Five CHX containers will be randomly chosen early, during and towards the end of the trial to check for CHX concentration and antibacterial activity. The chlorhexidine stock is securely kept below 30 °C, away from light in tightly closed/sealed bottles.

### Comparator

Participants in the comparison arm of the trial will be given the standard of care (dry cord care).

### Measurements

The main outcomes of this study are clinically diagnosed omphalitis and severe illness. Assessment for these outcomes is scheduled on days 1, 3, 7, 14 and 28 after birth. Further, the mothers are encouraged to visit our clinics if they observe any signs of umbilical cord stump infection or if they are worried that their child is otherwise ill. Severe illness is defined as illness that is associated with any of the following danger signs observed or verified by a study clinician: inability to feed or vomiting of all intake, lethargy or unconsciousness, severe lower chest in-drawing, axillary temperature of ≥37.5 °C or <35.5 °C, grunting, cyanosis, convulsions or a history of convulsions, and/or results in hospitalization and/or results in death. The data collection team records the presence or absence of omphalitis at each clinic visit. Signs for omphalitis include: pus, redness (inflammation) and swelling (edema) of the cord stump and the surrounding skin at its base. Swelling and redness is further broken down into four groups: none, mild, moderate and severe. No swelling or redness is defined as absence of visible swelling or redness; mild swelling or redness as that which is limited to the cord stump only; moderate swelling or redness as that extending less than 2 cm onto the abdominal skin at the base of the stump and severe swelling or redness as extending at least 2 cm or more around the abdominal skin at the base of the stump. Pus is characterized as being either present or absent. Cord infection is then defined based on combinations of these signs and their severity into four categories as follows: (1) redness extending to skin or pus, (2) moderate or severe redness, (3) moderate or severe redness with pus, or severe redness alone and (4) severe redness with pus [6, 7].

Given the necessity of the comparison arm to receive advice and education on dry cord care (the standard of care), this is an unblinded study. The study nurses are not made aware of the hypotheses that are being tested, and undergo rigorous training and standardization with respect to the assessment of omphalitis and severe illness to maintain a high validity in outcome assessment.

### Background characteristics and potential confounders

Using questionnaires and available records, data will be collected on several potential individual-level confounders. These include: maternal age, maternal education, antenatal care attendance, iron supplementation during pregnancy, antibiotic use during the last weeks of pregnancy, premature rupture of membranes, signs of chorioamnionitis, wealth, household size, parity, tetanus vaccination, gestational age, singleton or multiple birth, sex of the infant, birth weight, use of a clean delivery kit, prelacteal feeds, breastfeeding initiation time, infant’s receipt of colostrum, and Bacille Calmette-Guérin (BCG) vaccination. To further describe the population from which our participants are derived and to adjust for any contextual level confounders, we collect data using questionnaires on group/contextual-level factors such as the presence of electricity in the community, and residence (rural versus urban).

### Sample size

Sample size for primary objective 1: with type 1 error set at 5%, type 2 error at 0.2 (i.e., 80% power), and accounting for 5% attrition, a sample size of 4760 children will be required to detect a 30% relative reduction in the incidence of severe illness following a single cleansing of the umbilical cord stump with the 4% CHX solution. We assumed a 6% risk of severe illness in the control group.

Power calculation for objective 2: setting the type 1 error at 5% and accounting for 5% attrition, a total sample size of 4760 children will yield 96% power to detect a 30% relative reduction in the incidence of omphalitis following a single cleansing of the umbilical cord stump with the 4% CHX solution. The assumed risk of omphalitis in the dry cord care group (comparator) is 11.5% based on recently published data from the trial in Pemba [10].

### Data collection and management

#### Data collection: primary objectives

**Fig. 1:**
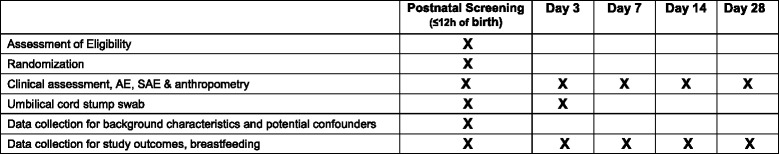
Time schedule for study procedures (Standard Protocol Items: Recommendations for Interventional Trials (SPIRIT) figure)

Our study staff in the three study clinics identifies HIV-1-negative pregnant women in the delivery rooms. Potential participants are approached and informed about the study. Following a live birth, study procedures are explained to the women, and babies of those providing informed consent are enrolled and then randomized to either the intervention or comparison arm by research assistants, using the cell phone-based application described above. For enrolled infants, data will be collected using questionnaires on day 1, and on days 3, 7, 14 and 28 after birth (Fig. 1).

Using a standardized approach (distance, light, etc.), at each of the above-defined visits and at any additional contact, data will be collected on umbilical cord stump infection and digital pictures are captured of the umbilical cord stump with its surrounding skin. Participating women will be invited to bring their children to the study clinics on the scheduled dates for data collection. Home visits will be conducted for women unable to come to the health facilities for the scheduled interviews (Fig. 1).

To capture severe illness, mothers and other caregivers will be informed about its symptoms and signs, and will be encouraged to contact the study clinic in case the baby develops any of the symptoms. Each mother is provided with a durable note book in which she or an attending health care worker are requested to note down all relevant events for the child. A child who comes to a clinic with such symptoms or signs will be examined by a study nurse/midwife and will receive the appropriate treatment. If the child does not attend after the mother/caregiver has informed the study team of such symptoms/signs or for a scheduled visit, the study nurse/midwife will make a visit to the child’s house. If the nurse/midwife classifies the condition as possible severe illness, a study physician will be called to examine the child. If the physician classifies the child as having severe illness, they will collect a blood specimen for blood culture (using BACTEC) and for a septic screen (C-reactive protein (CRP), total lymphocyte count, differential count, band cell: neutrophil count ratio) as soon as possible. A repeat specimen will be collected again for CRP between 24 and 48 h. The study team will do its utmost to obtain specimens for such a septic screen from as many infants with signs of severe illness as possible. With the consent of the parents, all children with neonatal sepsis are admitted to the National Referral and Teaching Hospital (Mulago Hospital) where they are managed according to the national guidelines. In the event of a neonatal death, it is our experience that a mother may call spontaneously to inform the study team of why she is not coming to the next scheduled visit. However, in most cases, she will simply not turn up; in that case, the death will be captured when she is contacted because of a missed visit. A “verbal autopsy” for all neonatal deaths will be conducted as soon as it is socially acceptable to maximize recall without socially offending the participants. We will assess the cause of death using a standard World Health Organization (WHO) Verbal Autopsy Questionnaire that has been validated in Uganda. The questionnaire has both open-ended questions (for verbatim narratives) and closed-ended questions, and we will employ a standard algorithm to determine the likely cause of death [12]. Two independent reviewers will examine the verbal autopsies and assign a likely cause of death. In event of divergent conclusions, a third reviewer will be requested to provide a third review.

### Project management

The study will be conducted as a collaboration between the College of Health Sciences, Makerere University and the Center for International Health, University of Bergen. The study will be guided by a Steering Committee which will include the principal investigator (PI) and the co-principal investigator (Co-PI) supported by the other co-investigators and a newly established Participatory Scientific Advisory Group (PSAG). The Steering Committee (VN and HS) is responsible for the conduct and coordination of the study. The Steering Committee will be the decision-making body for all scientific and administrative aspects. It will send reports to the funding agency, ethical committees and regulatory bodies. The SC will meet on regular conference calls with at least two face-to-face meetings annually. The study is registered with ClinicalTrials.gov (identifier: NCT02606565).

### Quality control

All data is collected using standardized questionnaires and forms on an ODK platform using Android cell phones. The study nurses were trained extensively in all the routines and how to fill the questionnaires and Case Report Forms before study implementation. In particular, they received training on the assessment and grading of omphalitis using audio-visual aids and pictorials by faculty members at the College of Health Sciences, Makerere University. Newborns with moderate to severe omphalitis or severe illness are immediately referred to the nearest government health unit. At least one scientist/medical officer is employed by the project to supervise the field team and ensure good-quality data. To minimize losses to follow-up, the following measures are taken: (1) careful screening of study participants for eligibility before enrollment, (2) consenting participants are provided with a transport refund that covers their costs to and from the health facility, (3) contact telephone numbers are obtained from each consenting participant and reminders are made prior to each scheduled date and (4) each study site has a dedicated tracer with good knowledge of the study site geography. Importantly, detailed information on the physical location/address of the participants’ homes is collected at enrollment to help with tracing mothers who do not attend for study visits. Research assistants (who are licensed nurses or midwives) have been, and are being, rigorously trained to obtain informed consent from study participates. They also receive training on all study tools which have been pretested and standardized to minimize information biases. The capture of digital images of the umbilical cord stump and surrounding skin will enhance the diagnostic specificity of reported omphalitis cases.

### Data analyses

Baseline characteristics of the mothers, their households and the newborns will be compared between the intervention and comparison arms to check for comparability and identify potential confounders. Continuous variables will be summarized using means, medians, standard deviations and interquartile ranges while percentages will be used for categorical variables.

**Fig. 2:**
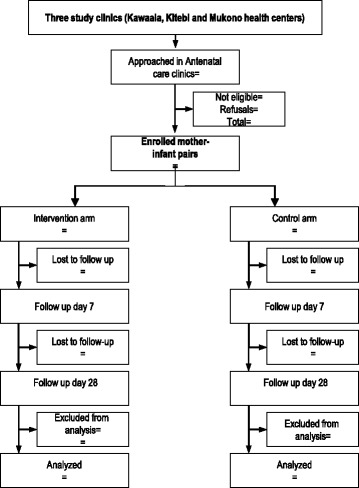
Flow chart of study participants

Analysis for both primary objectives will be based on intention-to-treat. Therefore, all infants randomized and enrolled into the study will be included in the final analysis regardless of whether CHX was applied to their umbilical cord stump and surrounding skin or not (Fig. 2: flow chart and Additional file 1: Standard Protocol Items: Recommendations for Interventional Trials (SPIRIT) Checklist). The risks of omphalitis and severe illness in the intervention arm will be compared to those in the comparison arm using binomial regression with a log link to obtain risk ratios (RR). Multivariable analysis will be used to take into account potential confounding. Only confounders whose inclusion in the model results in a substantial (>5%) change in the estimate of the main outcome will be maintained in the final model. Percent protective efficacy will be calculated as (1 − RR) × 100.

Subgroup analysis: subgroup analyses will be conducted. Associations between a single cord cleansing with 4% CHX and omphalitis will be stratified on relevant variables such as gestational age (±37 weeks (completed) of gestation) and birth weight (±2.5 kg). If substantial stratum-specific differences in effects are observed, we will proceed with analyses to identify effect measure modification. This may provide insight into possible interaction between the intervention and individual level as well as group- level contextual variables. Such effect measure modification analysis may provide potential causal explanations for our findings.

Equity impact analysis: further secondary analysis will compare the effectiveness of the intervention across strata of education levels, wealth quintiles and residence in order to assess the impact of the intervention on health and economic equity. All the data required for this analysis will be collected prospectively and, if the sample size allows, we shall estimate the effects of focusing this intervention on the most vulnerable groups. The Concentration Index, a bivariate measure of the distribution of important outcomes, will be used to measure any improved equity in outcomes.

## AE reporting/clinical and safety monitoring

In this study, adverse events (AEs) are defined as any harmful manifestations occurring in trial participants regardless of whether these manifestations are related or not to the application of CHX. Potential adverse events include localized chemical burns and delay in cord falling.

## AE monitoring, recording and reporting

At each study visit, potential AEs are being carefully examined for, and asked about, using study-specific questionnaires. In addition, mothers are advised to bring their babies to the study clinics in the event of any illness irrespective of the formal study visit schedule. At these walk-in visits, additional data is recorded on any AEs. AEs are recorded using medical terminology and, when possible, clinical diagnoses are recorded on the source document. The source document also includes the investigators’ opinion with regard to the relatedness of the AE to the CHX application. All neonates with severe adverse events (SAEs) will be followed up till resolution or till achievement of a stable clinical endpoint.

## AE reporting

An AE Reporting Form, based on the School of Medicine Research and Ethics Committee AE reporting template, is filled by a study investigator or physician, as soon as the study team becomes aware that a SAE has happened. A copy of the form will then be submitted to the Ethics Committee.

## Management of SAEs

Starting at enrollment, mothers are encouraged to bring their children to the study clinics immediately in the event of any serious illness. Once at the clinics, they are examined and referred for appropriate medical or surgical care at a government health facility. All illnesses reported at both scheduled and unscheduled visits are tracked and detailed information on duration, accessed health unit, treatment received and outcome is recorded.

## Monitoring

Independent Data Monitoring Committee (IDMC): three independent scientists with knowledge in statistics, epidemiology, pediatrics and obstetrics form the IDMC. The IDMC met in September 2016 and, thereafter, will meet (face to face or electronically) periodically to assess available study data for safety, conduct and efficacy. Subsequently, the IDMC will recommend the project management on study continuation, adjustment or termination based on its findings and pre-established stopping rules. An interim analysis for safety taking into account the DAMOCLES Group recommendations will be performed by the IDMC when about half of all the expected severe illness events have been reported [13, 14].

Monitoring: a site-readiness monitoring and audit visit from CISMAC (wwws.cismac.org) was done in August 2016; thereafter annual monitoring and auditing of the study will be conducted by a qualified scientist not participating in the day-to-day activities of the trial.

Protocol amendments: significant protocol adjustments will be communicated to, and discussed among, the SC members, the IDMC and the Ethics Committee.

## Ethics and dissemination

### Ethical considerations

Our study is an individually randomized trial in which half the participants will be randomized to receive a single application of 4% CHX on their umbilical cord stump and the other half will receive the government standard of dry cord care. There is a sufficient state of equipoise in Sub-Saharan Africa to justify this study because this intervention has never been tested, and only one study in Asia shows the effect of a single CHX application. Furthermore, two African trials [9, 10] that did not report on severe illness, found no effect of CHX on neonatal mortality, unlike the three Asian trials [6–8].

Four percent CHX has been found to be safe in community settings in Asia and Africa. Still, our study involves vulnerable human subjects living in a resource-constrained settings. Our study protocol follows the standard ethics review procedures and regulations in Uganda. All necessary approvals in Uganda, including those from the National Drug Authority have been obtained. In addition, an IDMC follows the trial and will report to the study sponsor. Participant data is strictly kept under lock and key or on password-protected computer databases for confidentiality.

### Dissemination and communication of results

Local communities and governments will be the key users of our study findings. To contribute to public access to research findings, we have already involved the Ugandan Ministry of Health and local communities and leaders in the conduct of the study. At the end of the study, community meetings will be convened at which research findings will be communicated. If the intervention is found effective and safe, we will, in collaboration with local leaders and other interested stakeholders, participate in the generation of health education materials, such as posters, that can be used to raise awareness and increase demand for the intervention. Similarly, officials at the Ugandan Ministry of Health, policy-makers and relevant NGOs will be apprised of the project and its results through meetings, reports, policy briefs, and other communication channels like email and telephone. We have interacted with the Department of Maternal, Newborn Child and Adolescent Health (MCA) at the WHO from the conception to the start of this trial. Such strong collaborations between the research team and decision and policy-makers will hasten the translation of research findings at the end of the study into policy and programs, locally, nationally and globally.

## Discussion

The current situation in which approximately 2.7 million children, mostly in LMICs, die within 28 days of birth is unconscionable. Uganda has one of the highest neonatal death rates in the world. Nearly 29 out of 1000 live-born children die within the first month of life. Sadly, this high risk of death in the first month of life in Uganda and in many other LMICs has been stagnant for decades and has not seen the same decline as for deaths of children aged between 1 and 5 years of age. Importantly, many of the neonatal deaths could possibly be prevented with affordable interventions such as 4% CHX. But results of the effect of 4% CHX applied to the umbilical cord stump on neonatal mortality in the three Asian trials are incongruent with findings in the two African trials. While all studies report an effect of 4% CHX on omphalitis, the African trials [9, 10] did not find an effect on neonatal mortality, unlike the Asian trials [6–8]. Moreover, none of these studies, particularly the African trials report on the potential effect of CHX on severe illness. It is, therefore, unclear whether the observed effect on omphalitis in the African trials translates into a protection from severe illness, an outcome that may be less prone to the Hawthorne effect than mortality. With multiple, sometimes daily, CHX applications and study visits by community health workers, the likelihood of obtaining a null finding because of this effect with a mortality outcome cannot be overlooked. This is particularly so in settings where frequent study visits have a high likelihood of coinciding with severe illness events which, in turn, result in interventions by the study team that likely lead to a reduction in mortality for all study arms. In addition, the effect of a single application of CHX has not been evaluated in SSA. If found effective in SSA, a single topical application of 4% CHX on the newborn umbilical cord stump has the potential to prevent hundreds of thousands of neonatal infections and deaths each year. This study is of particularly high relevance and benefit to societies in SSA where it will yield novel information because the effect of a single topical application of 4% CHX in birth facilities has not been studied, and where awareness and demand of this potentially life-saving intervention is nearly nonexistent among women, caregivers and health practitioners. Information produced from our trial will inform scale-up of the intervention if found effective in this setting.

### Trial status

Recruiting since July 2016.

## Additional file

Additional file 1: SPIRIT 2013 Checklist: recommended items to address in a clinical trial protocol and related documents*. (DOC 122 kb)

## Abbreviations

AE: Adverse event
CHX: Chlorhexidine
CISMAC: Centre for Intervention Science in Maternal And Child health
CRP: C-reactive protein
HIV: Human immunodeficiency virus
HU: HIV-uninfected
IDMC: Independent Data Monitoring Committee
LMICs: Low- and middle-income countries
RR: Risk ratio
SAE: Severe adverse events
WHO: World Health Organization
